# Optimization of spray dried yogurt and its application to prepare functional cookies

**DOI:** 10.3389/fnut.2023.1186469

**Published:** 2023-05-09

**Authors:** Anwar Ali, Muhammad Tasawar Javaid, Diana Tazeddinova, Ahmal Khan, Taha Mehany, Toshev Abduvali Djabarovich, Rabia Siddique, Waseem Khalid, Tayyaba Tariq, Wing-Fu Lai

**Affiliations:** ^1^Department of Epidemiology and Health Statistics, Xiangya School of Public Health, Central South University, Changsha, China; ^2^Department of Applied Biology and Chemical Technology, Hong Kong Polytechnic University, Kowloon, Hong Kong SAR, China; ^3^Food and Nutrition Society, Gilgit Baltistan, Pakistan; ^4^Department of Food Science, Government College University, Faisalabad, Pakistan; ^5^South Ural State University, Chelyabinsk, Russia; ^6^Food Technology Department, Arid Lands Cultivation Research Institute, City of Scientific Research and Technological Applications, Alexandria, Egypt; ^7^Department of Chemistry, Government College University Faisalabad, Faisalabad, Pakistan; ^8^University Institute of Food Science and Technology, The University of Lahore, Lahore, Pakistan; ^9^National Institute of Food Science and Technology, University of Agriculture, Faisalabad, Pakistan; ^10^Department of Urology, Zhejiang Provincial People's Hospital, Hangzhou Medical College, Hangzhou, China

**Keywords:** spray drying, yogurt, polyphenols, optimization, functional cookies

## Abstract

**Introduction:**

Spray-dried yogurt powder (SDYP) has shelf stability and other functional properties that improve solubility and facilitate the use, processing, packaging, and transportation of other food derivatives, such as bread and pastries on a large scale. The present research was conducted to develop SDYP and further its utilization to prepare functional cookies.

**Methods:**

Yogurt was spray-dried by employing different outlet air temperatures (OAT) (65°C, 70°C & 75°C) and inlet air temperature (IAT) (150°C, 155°C & 160°C). Spray drying shows that increasing the temperature increases nutritional loss, whereas *S. thermophilus* culture shows resistance to the intensive heat approaches. On the other hand *L. delbrueckii* subsp. Bulgaricus culture was found to be significantly affected. A total of 4 treatments, including one control for the functional cookies development.

**Results and discussion:**

A directly proportional relation was investigated between the increasing concentration of SDYP and baking characteristics and cookie's mineral and protein profile. Bioactive parameters like antioxidant activity of 2,2-diphenyl-1-picryl-hydrazyl-hydrate (DPPH), 2,2′-azino-bis (3-ethylbenzothiazoline-6-sulfonic acid (ABTS) and total phenolic content (TPC) were also affected significantly. The sensory profile shows an incline towards T0 (0% SDYP) to T3 (10% SDYP) in all attributes but starts to decline when the concentration of SDYP reaches 15%. This study suggests that by employing a certain combination of temperatures (OAT: 60°C IAT: 150°C); maximum survival of inoculated culture can be achieved, and this powder can be utilized in the development of functional cookies with enhanced sensory as well as biochemical characteristics significantly (*P*< 0.05).

## 1. Introduction

The term “yogurt” originates from the Turkish word “yogurmak” which is literally defined as coagulating, thickening or cuddling. For a millennium, yogurt has been a part of the human diet around the globe (1). The health benefits of yogurt go back to 6,000 BC; in the 20th century *Stamen Grigorov* a Bulgarian medical student reported on the health-promoting aspects and advantages of lactic acid bacteria (2, 3). Because yogurt is a good source of protein with excellent bioavailability, a rich source of calcium, and a source of a variety of health-promoting probiotics, low yogurt consumption deprives you of the opportunity to contribute to a healthier lifestyle (4–6).

The quantity of viable cells in yogurt powder is a valuable indicator for determining the severity of heat damage caused during drying and optimizing processing settings (7–9). Powder items must satisfy the requirement that they have more than 10^6^ colony-forming units (CFU)/ g at the expiration date to be considered healthful (7, 10). Because it has a high moisture removal rate, is less expensive, and takes less time to complete, spray drying is the most common and extensively researched alternative to freeze drying. It may be used for high throughputs and enables the preparation of stable and functioning products (11). The main issue with spray drying yogurt is keeping the lactic acid bacteria alive during and after drying (12). Spray drying must be light enough to prevent harming the heat-sensitive lactic acid bacteria while being effective enough to produce a powder with a moisture content below 4%, which is necessary for storage stability (13). Operating parameters for spray drying are primarily dependent on the equipment and the substance (14). The small range of potential operational parameters should be considered while optimizing spray-drying settings (15, 16).

The temperatures of the air entering and leaving the spray drier, the kind of atomization, and the airflow direction all impact the survival of yogurt bacteria. *Streptococcus thermophilus* was shown to have a greater survival rate than *Lactobacillus bulgaricus* during the spray drying of plain yogurt (17). However, both bacteria had comparable survival rates after the freezing process. The output temperature ranges of 70–75°C were ideal for *L. bulgaricus* and *S. thermophilus* survival; at these temperatures, the dried product's ultimate moisture content ranged from 5.1 to 6.3% (18). Therefore, the outlet air temperature during spray drying also significantly impacts the moisture content, color, and sensory characteristics of yogurt powder (19).

According to reports, fermented milk products have a hypocholesterolemic effect. Large amounts of fermented milk are recommended to offer factors that reduce cholesterol synthesis (20). *Lactobacillus acidophilus* has been reported to have the ability to reduce plasma cholesterol levels. Meanwhile, hypercholesterolemia is considered one of the leading causes of cardiovascular disease that can be treated with fermented dairy derivatives (20, 21). Dairy products have always been essential for human nutrition (18). However, further study of the product's anti-mutagenic, anti-cancer (22), and cholesterol-lowering properties will provide even greater opportunities for cultured dairy products, which are an essential component of human nutrition (23). However, to achieve the desired consumer benefits, it will be necessary to carefully select certain strains and combine them with appropriate production and processing procedures (24–26). Generally, probiotics and fermented foods are encouraged (27, 28).

Spray-dried milk powder has not only shelf stability but also other properties that improve solubility and facilitate the use, processing addition to packaging and transportation of other food derivatives such as bread and pastries on a large scale (29–32).

Cookies are one of the bakery products that all age groups people consume. These are usually prepared from refined wheat flour, which tends to have fewer essential nutrients but is a good source of fat, and carbohydrate (33, 34). The nutritional value can be enriched by dried yogurt powder, which adds protein, probiotics and minerals. The protein content of cookies can be increased by using yogurt powder (33, 35).

Spray drying can be employed for the drying of yogurt along with the highest survival of health-beneficial bacterial strains (36–38). SDYP can be an active functional ingredient for developing health-promoting food derivatives, which helps reduce health-related complications such as obesity. To develop SDYP, optimized processing conditions should be employed to achieve a certain derivative with acceptable probiotic bacteria viable count as well as maximum levels of protein content. There is no sufficient data available concerning the effect of spray drying on yogurt's physicochemical profile. However, yogurt powder is a great contender as a functional food constituent. It can be employed in developing functional cookies to enhance their nutritional value and bioactive profile. Taking all into context, the present study has been designed to evaluate the effect of drying yogurt by using the spray drying technique and further its application in developing functional cookies at different levels.

## 2. Materials and methods

### 2.1. Procurement of raw material

Raw material ( i.e., milk, wheat flour, sugar and fat etc.) was purchased from the local market of Faisalabad city in the highest possible quality. A total of 1,500 mL milk sample was collected in 3 sterilized glass bottles with having capacity of 500 mL each. Particularly milk samples were stored in a cool box (5 ± 2°C). Regents required in this effort were directly purchased from the scientific store of the highest possible quality. The collected raw material was transferred to the “Hi-Tech labs” facility at Government College University Faisalabad for further examination or development.

### 2.2. Development of yogurt

Milk samples were pasteurized before the inoculation of starter culture by employing high-temperature–short-time (HTST) technique with some modifications. For this purpose, accurately 200 mL of milk sample was taken in a graduated conical flask container of 500 mL capacity and further placed into a water bath (Model-1235 PC, *VWR scientific*, Singapore) and the water bath was allowed to heat up until required temperature (72°C) is achieved. After the pasteurization step, milk was placed in a glass jar, and starter culture comprising *L. bulgaricus* and *S. thermophilus* was introduced. The further fermentation process was carried out for about 8–12 h.

### 2.3. Spray drying of yogurt

Yogurt was stirred in a blender for 1 min (1st 20 s at low speed; 2nd 20 s high speed and 3rd 20 s again at low speed) and quickly heated up to the certain feed temperature before spray drying. Experiments were conducted in a pilot-scale spray dryer. Stirred yogurt was atomized from the nozzle into a vertical co-current drying chamber, 0.87 m diameter and 1.2 m in height, under various operating conditions. Atomizing air pressure of 296 kPa and hot air flow rate of 1.54 m^3^/m were fixed for all experiments. Inlet air temperature (150–160)°C, outlet air temperature (65–75)°C, and feed temperature 9°C were in the range of and were adjusted according to the central composite rotatable design (CCRD; Table 1). The outlet air temperature was controlled by regulating the feeding velocity. The dried powder was collected in a single cyclone separator, packaged in glass jars, and kept in the dark until used for analysis.

**Table 1:** Temperature optimization for spray drying of yogurt.

**Treatments**	**OAT (°C)**	**IAT (°C)**	**FT (°C)**
T1	65	150	9
T2	70	155	9
T3	75	160	9

OAT, outlet air temperature; IAT, inlet air temperature; FT, feed temperature.

### 2.4. SDYP analysis

#### 2.4.1. Culture survival

To determine survival rate of the lactic acid bacteria, dried samples were rehydrated to the initial solids level of fresh yogurt with distilled water. *S. thermophilus* and *L. bulgaricus* was enumerated according to TS ISO 7889. Ten grams of reconstituted yogurt powder was mixed with Ringer's solution and decimal dilutions were prepared. For the enumeration of *S. thermophilus*, M-17 agar plates were incubated aerobically at 37°C for 48 h and the double-layer inoculated MRS plates for *L. bulgaricus* were incubated anaerobically at 37°C for 72 h. The average counts from the plates of 30–300 colonies were calculated and the results were expressed in cfu/g dry matter.

#### 2.4.2. Moisture content

The moisture content of prepared cookies and dried apple pomace was used as prescribed by AOAC (39). Exactly, 2 g sample was taken into the crucible of known mass and placed into the oven for 3 h at 105°C. Further, the samples were cooled down in a desiccator and weighed using a digital balance. Obtained results were statistically analyzed and discussed in the fourth chapter. By the difference in mass, the mass of the moisture content in samples was calculated by the expression.

Calculations:


$$\begin{array}{l} Moisture\;content=\;\left(W_{2}-W_{3}\right)/\left(W_{2}-W_{1}\;\right)\times 100 \end{array}$$

where:

W1, weight of empty crucible; W2, weight of crucible + sample before drying; W3, weight of crucible + sample after drying to constant mass.

#### 2.4.3. Protein content in SDYP

The protein content of prepared SDYP was determined by utilizing Micro-Kjeldahl method as prescribed by AOAC (39). For this purpose, a conversion factor of 6.25 was employed to determine the total nitrogen. Accurately, 2 g of the sample was poured into the test tube, followed by 10 ml H_2_SO_4_ in the presence of selenium as a catalyst. However, digestion of the mixture continued until a clear solution appeared. After the digestion, the solution was transferred into a graded flask containing 100 mL of distilled water. Exactly, 10 mL of the prepared solution was mixed with equivalent volumes of NaOH (45%) solution and distilled by Kjeldahl apparatus. After distillation, the mixture was transferred into a 100 mL (4%) boric acid flask. Accurately, three drops of methyl red indicator and thoroughly mixed.

Moreover, 50 mL distillate was titrated against 0.02 N H_2_SO_4_ solution until the color changed from green to red. Obtained results were statistically analyzed. The expression calculated the nitrogen (%) content.

Calculations:


$$\begin{array}{l} Nitrogen\;\left(\%\right)=\left(V_{s}-V_{B}\times N_{A}\times 0.01401\right)/W\times 100\; \end{array}$$

where: VS, volume (ml) of acid required to titrate sample; VB, volume (ml) of acid required to titrate blank; NA, normality of acid; W, weight of samples in grams.


$$\begin{array}{l} \;\%\;crude\;protein\;=\;N2\;\times \;conversion\;factor\;\\ 100\%\;nitrogen\;in\;protein\;=\;conversion\;factor\;\\ \;100/16=6.25\; \end{array}$$

where: N2, nitrogen.

#### 2.4.4. pH determination

After the SDYP was rehydrated, it was analyzed for pH determination through a method prescribed by AOAC (40). Sample was thoroughly mixed and 15 g sample was taken in a china dish. After that, the pH. and temperature electrodes were wiped out with tissue paper and then placed in china dish. Both the readings of time and temperature were noted from the digital pH meter display screen. This method mentioned above was repeated for all samples (Temperature). The obtained data were statistically analyzed and stated in the results section.

*NOTE: After the above-mentioned analysis, the treatment with the least physicochemical loss and maximum survived (Culture) has been further employed in different concentrations to develop functional cookies*.

### 2.5. Development functional cookies

After the preparation of SDYP, it was added at different levels in the preparation of cookies, as shown in Table 2. The preparation steps were as follows: the ingredients (the whole wheat flour and dried apple pomace) have been used in a bowl. Further, fat, milk and salt were mixed for 30 min using the rubbing method. Kneading of dough was done by adding egg and water into the flour-based mixture in a separate bowl. In the next step, the dough was rolled and flattened into the similar thickness of about 3.5 mm before cutting into shapes using a hand cutter. The cut dough was baked in the oven at 150°C for ~30 min. After baking, the cookies were allowed to cool down at ambient room temperature and then packed in vacuumed low-density polyethylene bags to avoid contamination until further analysis.

**Table 2:** Different levels of SDYP in cookies.

**Treatments**	**Composition**	
	**Wheat flour (%)**	**SDYP (%)**
T0	100%	0%
T1	95%	5%
T2	90%	10%
T3	85%	15%

SDYP, spray-dried yogurt powder.

### 2.6. Proximate chemical analysis

Prepared SDYP and functional cookies samples were analyzed for their basic chemical profile i.e., moisture content, ash content, protein content, fat as well as fiber content and nitrogen free extract according to methods prescribed by AOAC (41). The calculations are as follows:


$$\begin{array}{l} \;Moisture\;content=\;\frac{W_{2}-W_{3}}{W_{2}-W_{1}}\times 100\;\\ Ash\;Content\;\left(\%\right)=\frac{\left(W1\right)\;Weight\;of\;Ash}{\left(W\right)\;Weight\;of\;initial\;sample}\times \frac{100}{1}\; \end{array}$$

$$\begin{array}{l} \;\frac{W_{2}-W_{1}}{Weight\;of\;sample}\times 100\;\\ Nitrogen\;(\%)=\frac{V_{s}-V_{B}\times N_{A}\times 0.01401}{W}\times 100\; \end{array}$$

100% Nitrogen in protein = conversion factor.


$$\begin{array}{l} \;\frac{100}{16}=6.25\;\\ Crude\;Fat\;(\%)=\frac{Mass\;of\;Fat}{Mass\;of\;Sample}\times 100\;\\ \;Crude\;Fiber\;(\%)=\frac{W_{1}-W_{2}}{W_{3}}\times 100.\; \end{array}$$

### 2.7. Bioactive profile of developed functional cookies

#### 2.7.1. Extraction of soluble phenolic compounds

For this purpose, samples were ground into a fine powder using a KMF grinder at 9,676.8 × g. Prepared ground samples were kept in sterile bags to prevent contamination at −40°C until further extraction. Methyl alcohol, ethyl alcohol and water were used to prepare extracts. Accurately, 0.5 g of dried sample was added into a flask followed by exactly 100 mL ethyl acetate and stirred at 20°C for 3 h. The prepared mixture was kept in the dark to avoid any exposure to light for this purpose, aluminum foil was employed; the mixture was held for 12 h. Next, mixture was centrifuged at 9,676.8 g for 30°C. Further, concentrate was filtered by Whatman filter paper (No 1, Ø 155 mm). However, prepared extracts were stored at ~4°C for until further analysis (42, 43).

#### 2.7.2. Antioxidant activity (DPPH assay)

Free radical scavenging activity was examined by method (2, 2-diphenyl-1-picryl hydrazyl) as prescribed by Mphahlele et al. (44) with slight modifications as mentioned above. For this purpose, accurately 15 μl extracts were added into a test tube followed by 735 μl methanol and 750 μL 0.1 mM DPPH solution and thoroughly mixed until the extract dissolved in methanol. Then the mixture was incubated for precisely 30 min in the dark to avoid any exposure to light. The absorbance was measured at 517 nm by employing Ultra Violet visible spectrophotometer. A suitable calibration curve was prepared by using ascorbic acid as standard solution. The results were expressed as mM ascorbic acid (AA) equivalent g^−1^ of extracts.

#### 2.7.3. Total phenolics content

In this study, prepared extracts were examined for their total phenolics content (TPC) by Folin-Ciocalteu method as prescribed by Al-Rawahi et al. (45). For this purpose, accurately 70 μL of prepared extracts were added in a test tube of 10 ml capacity, followed by 250 μl of Folin-Ciocalteu reagent and 750 μL of Na_2_CO_3_ (1.9 M). However, a total volume of exactly, 5 m was made up by adding distilled water and then mixed by using a vortex mixer for about 1 min prior to incubation for 2 h. in the dark. Consequently, the absorbance was measured using spectrophotometer (Thermo-Spectronic, Surrey, England) at 765 nm wavelength. Calibration curve was prepared by employing controlled solutions of gallic acid. Obtained results were expressed as gallic acid equivalents (GAE) in mg^−*g*^ dry solids.

#### 2.7.4. Total flavonoids content

Total flavonoids content (TFC) content of the extract was determined by a method as prescribed by Al-Rawahi et al. (45). For this purpose, exactly 1 mL of prepared extract was placed into a test tube (10 mL) already containing 4 mL of distilled water. At instant, 0.3 mL of 5% sodium nitrite was added into the test tube. However, after 5 min accurately, 0.3 mL of 10% Aluminum chloride was also placed in the same test tube. Then after 6 min exactly, 2 mL of 1 M sodium hydroxide was added to the test tube and mixed. Instantly, the test tube was diluted with the addition of 2.4 mL of distilled water and thoroughly mixed.

At last, the absorbance of the pink-colored mixture was examined at 510 nm and water was used as a blank. A suitable calibration curve was prepared to utilize different concentrations of catechin solutions. The results were mg catechin equivalent (CE)/g of dry solids.

### 2.8. Sensory evaluation

Sensory evaluation of functional cookie samples was done using a 9-point hedonic scale. Sensory attributes were judged by a panel of trained judges relevant to the field of study. The parameters on the scale were as follows: 1 = dislike, 2 = dislike slightly, 3 = neither like or dislike, 4 = like moderately, 5 = like very much, 6 = like extremely, 7 = good, 8 = very good, 9 = excellent. Scores given by the judges were statistically analyzed (descriptives and ANOVA).

### 2.9. Statistical analysis

The obtained results were subjected to the analysis of variance (ANOVA) using the Statistical Package for Social Sciences (SPSS) Version 25 and the treatment means were separated using Fishers Less Significant difference (LSD) test.

## 3. Results

### 3.1. Effect of spray drying on the physicochemical profile of yogurt powder

**Figure 1:**
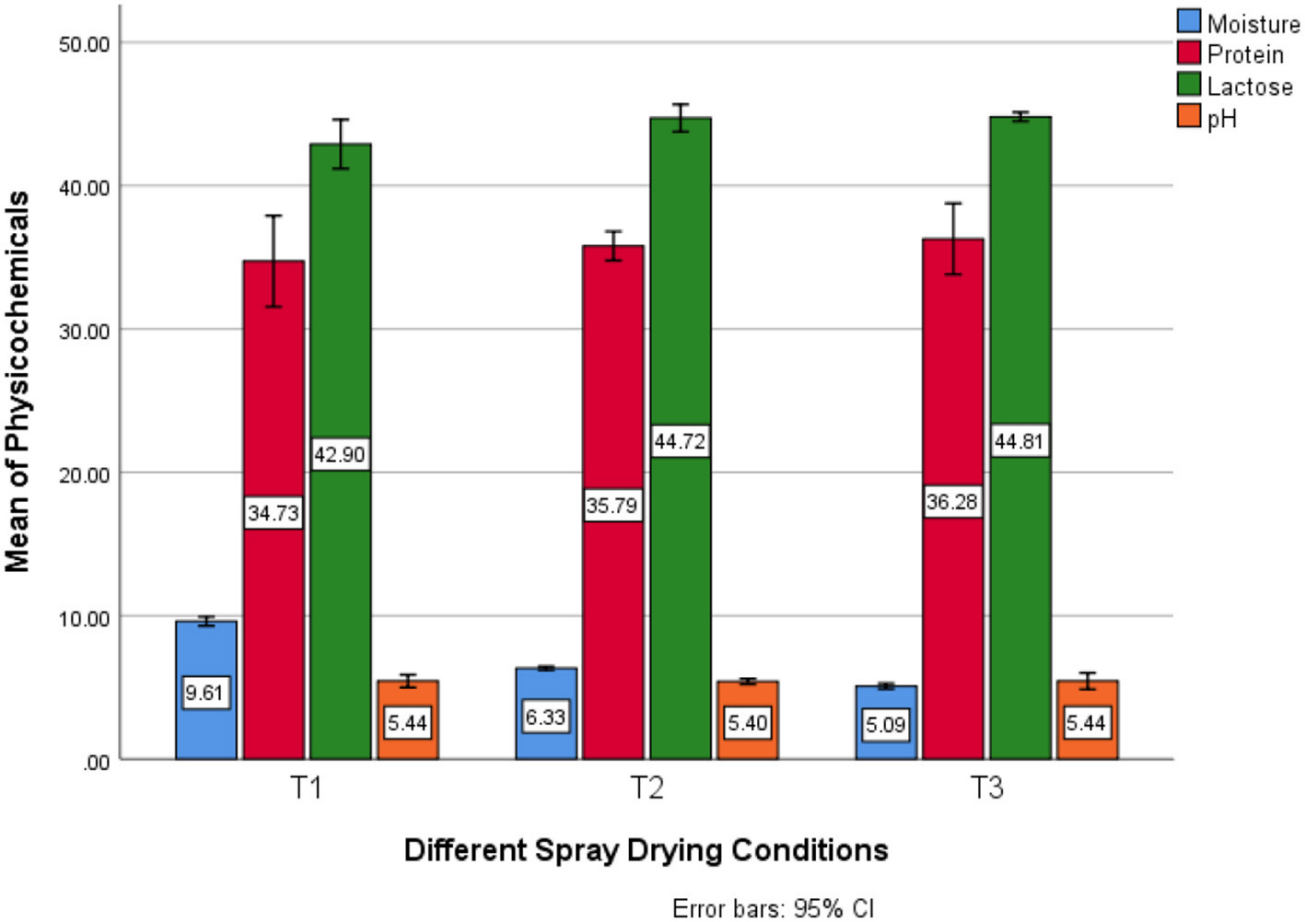
Estimated marginal means for physicochemical profile of SDYP.

As shown in Table 3, after the development of yogurt powder, samples were examined for their moisture; on average in T1 (9.60 ± 0.03) g/100 g, T2 (6.33 ± 0.01) g/100 g and in T3 (5.08 ± 0.02) g/100 g was found. Meanwhile, the highest water content was discovered in T1 (9.60) g/100 g samples treated with 65°C outlet temperature and 150°C of inlet air temperature. On the other hand, the lowest moisture content was investigated in T3 (5.08) samples being treated with 75°C outlet temperature and 160°C of inlet air temperature. Regarding the protein results, on average, in T1 (34.73 ± 0.35) g/100 g, T2 (35.79 ± 0.11) g/100 g and T3 (36.28 ± 0.27) g/100 g were found; meanwhile, the highest protein content was discovered in T3 (36.28) g/100 g samples treated with 75°C outlet temperature coupled with 160°C of inlet air temperature, on the other hand, least protein content was calculated in T1 (5.08) g/100 g samples being treated with 65°C outlet temperature coupled with 150°C of inlet air temperature. Interestingly, on average lactose value in T1 (42.89 ± 0.19) g/100 g, T2 (44.71 ± 0.11) g/100 g and in T3 (44.80 ± 0.03) g/100 g was found; meanwhile, the highest protein content was discovered in T3 (44.80) g/100 g samples treated with 75°C outlet temperature coupled with 160°C of inlet air temperature, on the other hand, least lactose content was calculated in T1 (42.89) g/100 g samples being treated with 65°C outlet temperature coupled with 150°C of inlet air temperature. As far as the pH determination was concerned it was clearly shown in Table 1. That on average, in T1 (5.43 ± 0.05), T2 (5.40 ± 0.02) and T3 (5.43 ± 0.06) were found; meanwhile, the highest pH value was defined in T3 (5.44) samples treated with 75°C outlet temperature coupled with 160°C of inlet air temperature whereas, minimum pH value was measured in T2 (5.40) samples being treated with 65°C outlet temperature coupled with 150°C of inlet air temperature (Figure 1).

**Table 3:** Proximate chemical analysis of spray dried powder at different optimized conditions.

**Trials**	**Physicochemical profile**		
	**Moisture content**	**Protein**	**Lactose**	**pH**
T1	9.60 ± 0.03a	34.73 ± 0.35c	42.89 ± 0.19b	5.43 ± 0.05a
T2	6.33 ± 0.01b	35.79 ± 0.11b	44.71 ± 0.11a	5.40 ± 0.02b
T3	5.08 ± 0.02c	36.28 ± 0.27a	44.80 ± 0.03a	5.43 ± 0.06a
Average	7.00 ± 2.08	35.60 ± 0.74	44.14 ± 0.96	5.42 ± 0.04

T1, OAT 65°C: IAT 150°C: FT 9°C; T2, OAT 70°C: IAT 155°C: FT 9°C; T3, OAT75°C: IAT 160°C: FT 9°C.

Each value is an average of three observations.

Means that do not share a letter are significantly different at level (P < 0.05).

### 3.2. Effect of different spray drying conditions on survival of inoculated culture

**Figure 2:**
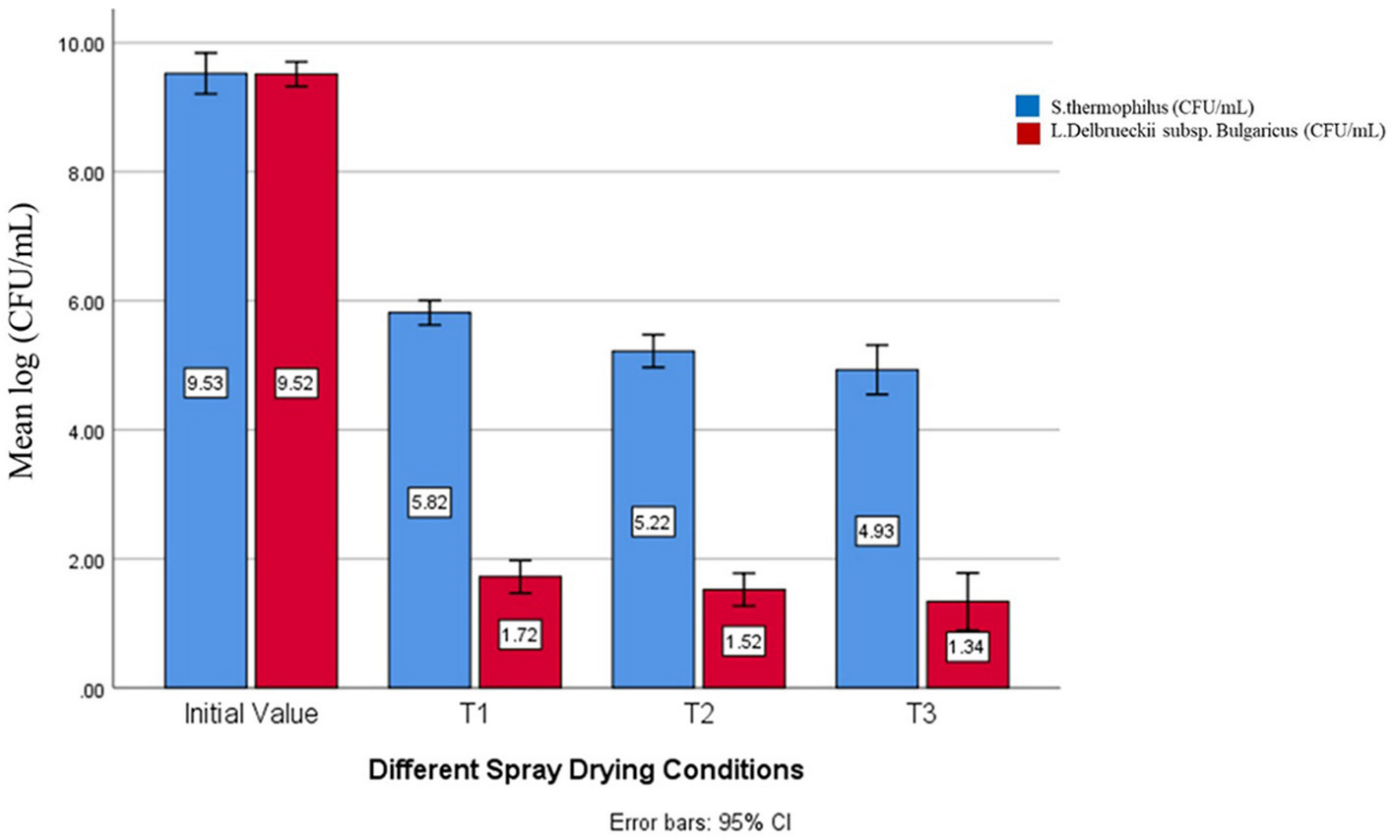
Estimated marginal means for culture survival in SDYP.

Two different strains were added for fermentations A: *S. thermophilus* and B: *Lactobacillus delbrueckii* subsp. *bulgaricus* in the current venture. Further, after each treatment (T0, T1, T2, and T3) yogurt powder was examined for both A and B strains which are depicted from (Table 6) on average in the initial sample 9.5 × 10^8^ log cfu/mL was detected for both A and B strains as far the A culture reduction was concerned, on average in T1 (5.8 × 10^8^) log cfu/mL (61% survival), T2 (5.2 × 10^8^) log cfu/mL (54.17% survival) and in T3 (4.9 × 10^8^) log cfu/mL (51.6% survival) was investigated. On the other hand, in culture B: on average in T1 (1.7 × 10^8^) log cfu/mL (17.9% survival), T2 (1.5 × 10^8^) log cfu/mL (15.8% survival) and in T3 (1.3 × 10^8^) log cfu/mL (13.7% survival) was found as shown in Table 4 and Figure 2.

**Table 4:** Effect of different spray drying parameters on culture survival.

**Trials**	**Inoculated culture**		
	**A**	**Survival%**	**B**	**Survival%**
Initial value	9.5 × 10^8^	NA	9.5 × 10^8^	NA
T1	5.8 × 10^8^	61.1	1.7 × 10^8^	17.9
T2	5.2 × 10^8^	54.17	1.5 × 10^8^	15.8
T3	4.9 × 10^8^	51.6	1.3 × 10^8^	13.7
Average	6.37 × 10^8^		3.52 × 10^8^	

T1, OAT 65°C: IAT 150°C: FT 9°C; T2, OAT 70°C: IAT 155°C: FT 9°C; T3, OAT75°C: IAT 160°C: FT 9°C; A, Streptococcus thermophilus (cfu/mL); B, Lactobacillus delbrueckii subsp. bulgaricus (cfu/mL); NA, not applicable.

### 3.3. Effect of adding SDYP on the proximate chemicals of functional cookies

After SDYP, cookies were prepared by partially substituting wheat flour with yogurt powder to enhance their bioactive profile, biochemical composition, and the desired sensory appeal. For this purpose, four treatments (T0: 0% SDYP, T1: 5% SDYP, T2: 10% SDYP and T3: 15% SDYP), including one control, were introduced in this study. However, the obtained results of its biochemical and bioactive and sensory profile were statistically analyzed as follows: Descriptive analysis of moisture content in different treatments was conducted. The maximum moisture content (5.21) g/100 g was detected in T3 sample with 15% SDYP whereas the least moisture level was examined in T0 (Control) g/100 g sample containing 0% SDYP. Examination shows that by adding SDYP moisture content of cookies significantly increased as compare to the cookies developed with only wheat flour and further found in following order (Wheat: SDYP) of T0 (100:0) < *T1* (95:05) < *T2* (90:10) < *T3* (75:15) at level (*P* < 0.05). Protein content in different treatments were conducted which clearly shows that the maximum protein content (16.22) g/100 g was detected in T3 sample having 15% SDYP whereas, least protein level (10.45) was examined in T0 (Control) g/100 g sample containing 0% SDYP. Analysis clearly shows that by adding SDYP protein content of cookies significantly increased as compare to the cookies developed with only wheat flour and further found in following order (Wheat: SDYP) of T0 (100:0) < *T1* (95:05) < *T2* (90:10) < *T3* (75:15) at level (*P* < 0.05). Fat content in different treatments was examined. Regarding the outcomes, determination shows that by adding SDYP fat content of cookies significantly decreased as compare to the cookies developed with only wheat flour and further found in following order (Wheat: SDYP) of T0 (100:0) > *T1* (95:05) > *T2* (90:10) > *T3* (75:15) at level (*P* < 0.05). While fiber and ash content were found to be increased but not significantly (Table 5).

**Table 5:** Effect of adding SDYP on the physicochemical profile of cookies.

**Descriptives (g/100 g)**						
**Treatments**	**Moisture**	**Protein**	**Fat**	**Fiber**	**Ash**	**Nitrogen free extract (NFE)**
T0	4.69 ± 0.26d	10.46 ± 0.01d	22.82 ± 0.03a	0.42 ± 0.03a	1.43 ± 0.01d	60.19 ± 0.19a
T1	4.96 ± 0.04c	12.09 ± 0.02c	22.14 ± 0.02b	0.41 ± 0.02a	1.44 ± 0.02c	58.96 ± 0.10b
T2	5.07 ± 0.04b	14.75 ± 0.04b	21.89 ± 0.04c	038 ± 0.04b	1.46 ± 0.01b	56.45 ± 0.12c
T3	5.20 ± 0.02a	16.19 ± 0.03a	20.98 ± 0.01d	0.35 ± 0.01c	1.48 ± 0.01a	55.80 ± 0.08d
Average	4.98 ± 0.22	13.37 ± 2.39	21.95 ± 0.70	0.39 ± 0.02	1.45 ± 0.02	57.85 ± 1.92

T0, control (100% wheat flour: 0% SDYP); T1, control (95% wheat flour: 5% SDYP); T2, control (90% wheat flour: 10% SDYP); T3, control (85% wheat flour: 15% SDYP).

Each Value is an average of three observations.

Means that do not share a letter are significantly different at level (P < 0.05).

### 3.4. Effect of adding SDYP on the baking attributes of functional cookies

**Figure 3:**
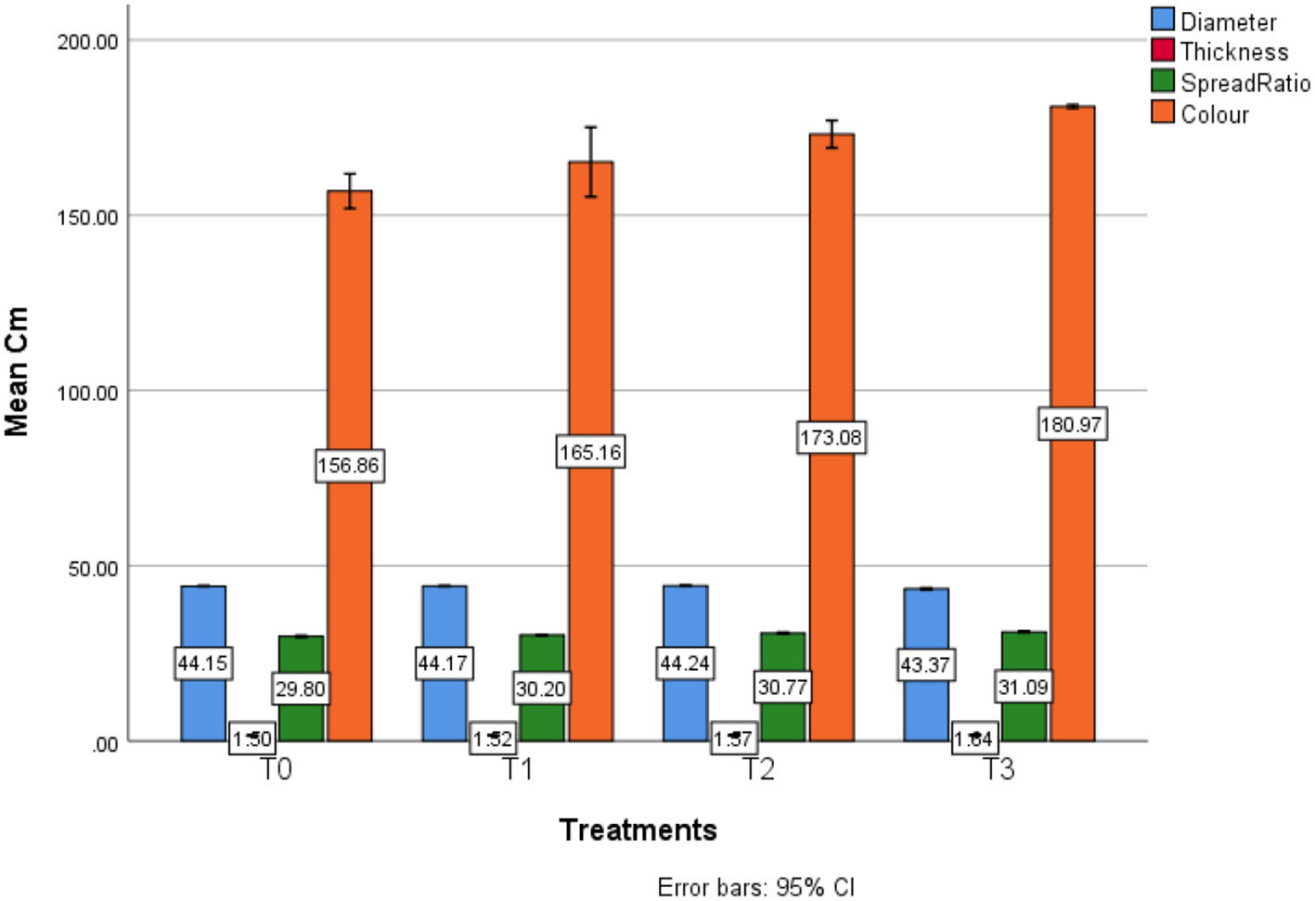
Estimated marginal means for baking profile in different treatments.

Descriptive analysis of the physical profile of different treatments was conducted. Regarding the diameter attribute results concerned, on average, in T0 (44.15 ± 0.01) mm, T1 (44.17 ± 0.01) mm, T2 (44.24 ± 0.01) mm, and T3 (43.37 ± 0.03) mm was investigated. The maximum diameter value (44.25) mm was calculated in T2 sample having 10% SDYP, whereas the least diameter value was examined in T3 sample (43.35) mm containing 15% SDYP. Thickness in T0 (1.50 ± 0.01) mm, T1 (1.52 ± 0.01) mm, T2 (1.57 ± 0.01) mm, and T3 (1.64 ± 0.01) mm was investigated. Whereas the maximum diameter value (9.27) mm was calculated in T3 sample having 15% SDYP whereas least thickness level was examined in T0 (control) sample (1.49) mm containing 0% SDYP. Regarding the spread ratio attribute results are concerned, on average T0 (29.80 ± 0.03), T1 (30.20 ± 0.01), T2 (30.77 ± 0.02) and T3 (31.09 ± 0.02) was investigated. The maximum spread ratio value (31.10) was calculated in T3 sample having 15% SDYP, whereas the least spread ratio was measured in T0 sample (29.78) containing 0% SDYP. Regarding the color attribute results concerned, on average T0 (156.86 ± 0.55), T1 (165.16 ± 1.10), T2 (173.08 ± 0.44) and T3 (180.97 ± 0.06) were investigated. The maximum color (181.01) was observed in T3 sample having 15% SDYP. In contrast, least color value was examined in T0 sample (156.47) containing absolutely 0% SDYP, as shown in Table 6 and Figure 3.

**Table 6:** Effect of adding SDYP on the baking characteristics of cookies.

**Descriptives**				
**Attributes**				
**Treatments**	**Diameter (mm)**	**Thickness (mm)**	**Spread ratio (D/T)**	**Color**
T0	44.15 ± 0.01a	1.50 ± 0.01c	29.80 ± 0.03d	156.86 ± 0.55d
T1	44.17 ± 0.01a	1.52 ± 0.01c	30.20 ± 0.01c	165.16 ± 1.10c
T2	44.24 ± 0.01b	1.57 ± 0.01b	30.77 ± 0.02b	173.08 ± 0.44b
T3	43.37 ± 0.03c	1.64 ± 0.01a	31.09 ± 0.02a	180.97 ± 0.06a
Average	43.98 ± 0.38	1.55 ± 0.06	30.46 ± 0.53	169.02 ± 9.61

T0, control (100% wheat flour: 0% SDYP); T1, control (95% wheat flour: 5% SDYP); T2, control (90% wheat flour: 10% SDYP); T3, control (85% wheat flour: 15% SDYP).

Each Value is an average of three observations.

Means that do not share a letter are significantly different at level (P < 0.05).

### 3.5. Effect of adding SDYP on bioactive profile of functional cookies

**Figure 4:**
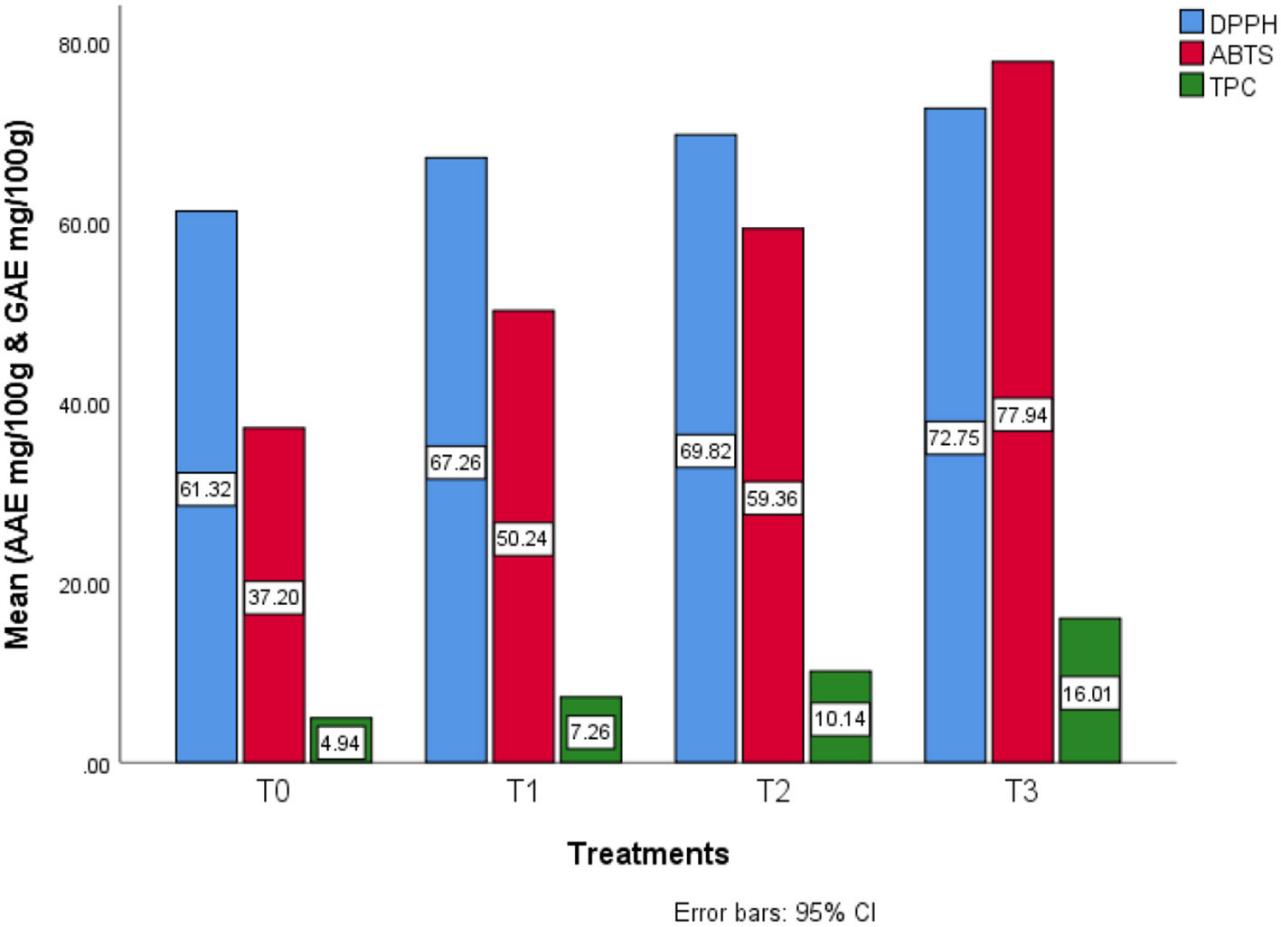
Estimated marginal means for bioactive profile in different treatments.

As shown in Table 7, descriptive analysis of DPPH inhibition in different Treatments were analyzed. As far the results are concerned, on average in T0 (61.30 ± 0.01) AAE mg/100 g, T1 (67.18 ± 0.01) AAE mg/100 g, T2 (69.93 ± 0.28) AAE mg/100 g and in T3 (72.80 ± 0.16) AAE mg/100 g was observed. The maximum DPPH inhibition (72.91) AAE mg/100 g, also known as antioxidant activity, was found in T3 sample having 15% SDYP whereas minimum DPPH inhibition was calculated in control (61.29) AAE mg/100 g samples developed from absolutely no SDYP. Descriptive analysis of ABTS inhibition in different treatments were analyzed. As far the results are concerned, on average in T0 (37.34 ± 0.21) AAE mg/100 g, T1 (50.26 ± 0.19) AAE mg/100 g, T2 (59.67 ± 0.49) AAE mg/100 g, and in T3 (78.14 ± 0.33) AAE mg/100 g was observed. The maximum ABTS inhibition (78.37) AAE mg/100 g also known as antioxidant activity was found in T3 sample having 15% SDYP whereas, minimum ABTS inhibition was calculated in control (37.19) AAE mg/100 g samples developed from absolutely no SDYP. Descriptive analysis of TPC in different treatments was analyzed. As far as the results are concerned, on average, in T0 (4.95 ± 0.02) GAE mg/100 g, T1 (7.30 ± 0.07) GAE mg/100 g, T2 (10.22 ± 0.13) GAE mg/100 g, and in T3 (16.18 ± 0.28) GAE mg/100 g was observed. The maximum TPC content (16.37) GAE mg/100 g, also known as phenolics, was found in the T3 sample having 15% SDYP. In contrast, the minimum TPC was calculated in control (4.93) GAE mg/100 g samples developed from absolutely no SDYP. In this current study, by partially adding SDYP in the development of functional bread; overall ABTS, DPPH inhibition as well as TPC content was found significant increase in the following order (Wheat: SDYP) of T0 (100:0) < *T1* (95:05) < *T2* (90:10) < *T3* (85:15) at level (*P* < 0.05; Figure 4).

**Table 7:** Effect of adding SDYP powder on bioactive profile of cookies.

**Descriptives**			
**Bioactive parameters**			
**Treatments**	**DPPH (%)**	**ABTS (%)**	**TPC (GAE mg/100 g)**
T0	61.32 ± 0.04d	37.20 ± 0.01d	4.94 ± 0.01d
T1	67.26 ± 0.13c	50.24 ± 0.16c	7.26 ± 0.01c
T2	69.82 ± 0.13b	59.36 ± 0.05b	10.14 ± 0.03b
T3	72.75 ± 0.08a	77.95 ± 0.05a	16.01 ± 0.04a
Average	67.79 ± 4.50	56.18 ± 15.85	9.59 ± 4.42

T0, control (100% wheat flour: 0% SDYP); T1, control (95% wheat flour: 5% SDYP); T2, control (90% wheat flour: 10% SDYP); T3, control (85% wheat flour: 15% SDYP).

Each value is an average of three observations.

Means that do not share a letter are significantly different at level (P < 0.05).

### 3.6. Effect of adding SDYP on the sensory profile of functional cookies

**Figure 5:**
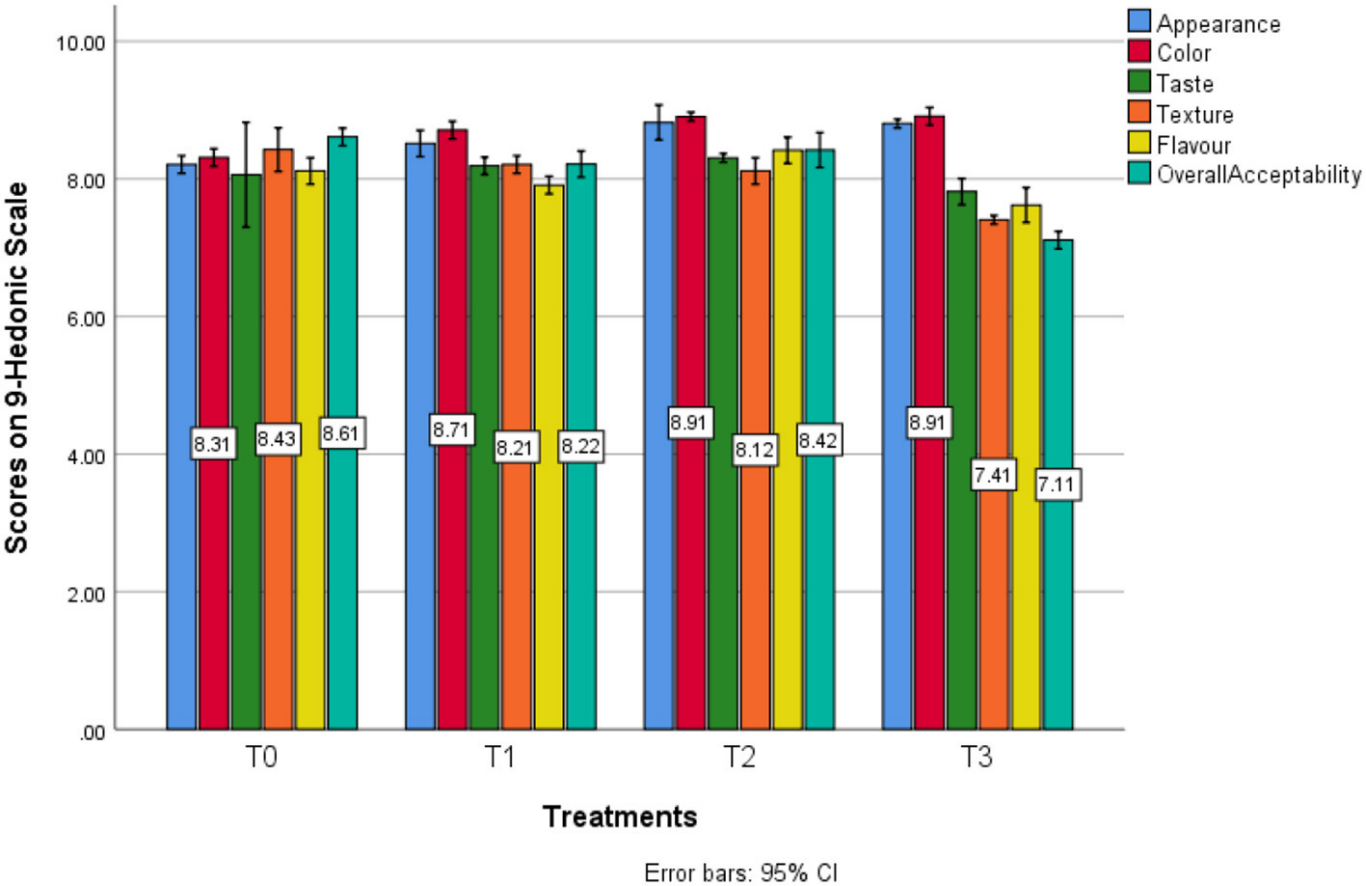
Estimated marginal means for sensory profile in different treatments.

On average, scores for appearance profile examination depicts that the maximum (8.8) score for color was observed in T3 (15% SDYP) samples as far least (8.2) score was calculated in T0 (control) samples. Although, flavor profile examination depicts that the maximum (8.4) score for flavor was observed in T2 (10% SDYP) samples as far least (7.6) score was calculated in T3 (15% SDYP) samples. Moreover, the taste profile investigation depicts that the maximum (8.3) score for taste was defined in T2 (10% SDYP) samples as far least (7.8) score was calculated in T3 (15% SDYP) samples. On average, scores for color in T0 (8.3), T1 (8.7), T2 (8.8), and T3 (8.9) were detected. Although, color profile examination depicts that the maximum (8.9) score for color was defined in T3 (15% SDYP) samples as far least (8.3) score was measured in T0 (0% SDYP) samples. By adding SDYP concentrations in the development of cookies, the overall acceptability was increased gradually (Figure 5). Meanwhile, results from T2 having 10% SDYP levels, respectively exhibits that SDYP can be added in certain amounts as discussed earlier to fulfill the nutritional gap generated by typical wheat cookies primarily prepared with wheat flour while without losing any of its sensory attributes at level (*P* < 0.05; Table 8).

**Table 8:** Effect of adding SDYP on the sensory profile of cookies.

**Sample**	**Appearance**	**Color**	**Taste**	**Texture**	**Flavor**	**Overall acceptability**
T0	8.2c	8.3c	8.0b	8.4a	8.1b	8.6a
T1	8.5b	8.7b	8.2a	8.2b	7.9c	8.2c
T2	8.7a	8.8a	8.3a	8.1b	8.4a	8.4b
T3	8.8a	8.9a	7.8c	7.4c	7.6d	7.1d

T0, control (100% wheat flour: 0% SDYP); T1, control (95% wheat flour: 5% SDYP); T2, control (90% wheat flour: 10% SDYP); T3, control (85% wheat flour: 15% SDYP).

Each value is an average of three observations.

Means that do not share a letter are significantly different at level (P < 0.05).

## 4. Discussion

During the spray drying of yogurt, it was observed that by increasing the outlet and inlet temperatures while keeping feed temperature constant, protein content and lactose content in dried yogurt powder tend to increase significantly. In contrast, a reduction in moisture content was investigated. In addition, there was no significant effect on pH readings examined. These results were found in engagement with the outcomes reported by Bielecka and Majkowska (18), Fang et al. (46), Guerin et al. (47), and Lam and Nickerson (48). Protein is a heat-sensitive counterpart of a food system; meanwhile, particularly in milk, it becomes more apparent; Lam et al. (48) explained that increasing protein and lactose at constant feeding is mainly due to the rapid evaporation coupled with less contact time with heat source. Effect of drying on the survival of inoculated culture was also examined, which shows that by increasing the outlet and inlet temperatures while keeping feed temperature constant, culture survival in dried yogurt powder tends to decrease significantly, as stated by Barbosa et al. (49).

In contrast, culture B (*L. delbrueckii* subsp. *bulgaricus*) was found significantly affected by the heat treatments whereas, culture A (*S. thermophilus*) was found to resist the heat to an extent. Low temperature (60°C) had a negative effect on powder texture (excessive moisture causing powder lumping). Considering sensory properties, moisture content, and viability of yogurt culture, it was concluded that the outlet air temperature of 70–75°C is optimal for spray drying yogurt. The effect of moisture content of yogurt powder on shelf life during storage will be the subject of further research. Further, a study conducted by Bielecka and Majkowska (18), Rolfe and Daryaei (50), and Khalid et al. (51) explaind that several factors such as the phase of bacterial growth and water activity of dried material along with potential acidity can affect the survival of thermophilic lactic acid bacteria. These results were relevant to the outcomes reported by Bielecka and Majkowska (18), Fang et al. (46), Guerin et al. (47), and Lam and Nickerson (48). A directly proportional relation was investigated between the increasing concentration of SDYP and baking characteristics of cookies (52). Meanwhile, by incorporating SDYP bioactive parameters such as DPPH, the ABTS and TPC count increased significantly (5). The sensory profile shows an incline toward T0 (0% SDYP) to T3 (10% SDYP) in all attributes, i.e., color, flavors, taste, and overall acceptability. Still, it starts to decline when the concentration of SDYP reaches 15%. An inversely proportional trend was observed that increased SDYP concentrations in functional cookies; baking characteristics tended to increase significantly (53). However, these means were studied at confidence level of 95%. In addition, by partially adding SDYP in the development of functional cookies; overall TPC, DPPH and ABTS was found significantly increased in following order of (Wheat: SDYP) of T0 (100:0) < *T1* (95:05) < *T2* (90:10) < *T3* (85:15) at level (*P* < 0.05). It was observed that by adding SDYP concentrations in the development of cookies, the acceptability found to be increased gradually. Meanwhile, results from T2 having 10% SDYP powder levels, respectively exhibits that SDYP can be added in certain amounts as discussed earlier to fulfill the nutritional gap generated by typical wheat cookies primarily prepared with wheat flour while without losing any of its sensory attributes at level (*P* < 0.05).

## 5. Conclusion

Spray drying is a common method used in the food processing industry to acquire distinctive properties, such as instant solubility and to improve product shelf stability. It is considered the foremost drying approach of the technology used in dairy products due to its low operating costs and high production rates. In this study, yogurt was spray dried using several optimum OAT (65, 70, and 75°C) and IAT (150, 155, and 160°C) settings. Spray drying indicates unequivocally that nutritional loss tends to increase as temperature rises. *Streptococcus thermophilus* culture, on the other hand, exhibits resilience to intense heat approaches, while *L. delbrueckii* subsp. *bulgaricus* culture was found to be considerably impacted. A total of four treatments, including one control, were used to create functioning cookies. The relationship between rising SDYP content and baking properties and cookies' mineral and protein profile was directly proportional. Bioactive measures like DPPH, ABTS, and TPC count were also considerably impacted. The sensory profile shows an incline toward T0 (0% SDYP) to T3 (10% SDYP) in all attributes but starts to decline when the concentration of SDYP reaches 15%. The practical application of this study suggests that by employing a certain combination of temperatures (OAT: 60°C) and (IAT: 150°C), the maximum survival of inoculated culture can be achieved and this powder can be utilized in the development of functional cookies with enhanced sensory as well as biochemical characteristics.

## Data availability statement

The raw data supporting the conclusions of this article will be made available by the authors, without undue reservation.

## Author contributions

AA: conceptualization. AA, MTJ, DT, and AK: writing-original draft preparation. MTJ, DT, TM, TAD, RS, WK, and TT: writing-review and editing. W-FL: supervision. All authors have read and agreed to the published version of the manuscript.
